# Reversible data hiding and authentication scheme for encrypted image based on prediction error compression

**DOI:** 10.1038/s41598-025-95433-9

**Published:** 2025-04-04

**Authors:** Fang Ren, Zhelin Zhang, Kai Jiang, Peiyang Zhang, Tengfei Yang

**Affiliations:** 1 School of Cyberspace Security, Xi’an University of Posts and Telecommunications, Xi’an, 710121 China; 2 National Engineering Research Center for Secured Wireless, Xi’an, 710121 China

**Keywords:** Reversible data hiding, Encrypted image, Authentication, Prediction error, Computer science, Information technology

## Abstract

In the most existing reversible data hiding schemes for encrypted images, cover images can be reversibly recovered, but the integrity of image content cannot be guaranteed. This paper proposes a reversible data hiding and authentication scheme for encrypted images to implement reversible recovery and content authentication of cover images and secret data. The content owner employs a new predictor ISGAP to generate more accurate predictions and smaller errors. The errors are then compressed with adaptive Huffman coding to enlarge the embedding space, and the plaintext authentication information is embedded in it. The data hider embeds secret data into the encrypted image along with ciphertext authentication information. The receiver performs ciphertext authentication first and then implements cover recovery and plaintext authentication according to different keys. Experiments were carried out with 100 images selected from each dataset of BOSSbase and BOWS-2, and the results show that the scheme has higher embedding capacity and can effectively implement image content authentication while ensuring high security and reversible recovery.

## Introduction

Data hiding^1–3^ is a technology that realizes the covert transmission by embedding secret data into carriers such as text, video, and images. With the development of computer and Internet technology, the application of digital images is becoming more extensive. Massive digital images are used in various websites, instant messaging, e-mail and so on. There have been many achievements in the research of image-based data hiding. Some schemes use images as carriers to achieve covert transmission, and some schemes achieve protecting the integrity of images by embedding authentication information. There exist scenarios where the unauthorized dissemination of the image, or its role as a conveyance medium, can profoundly affect the communicating parties, particularly within sensitive areas such as military operations and healthcare. These applications emphasize the necessity not only of transmitting confidential data discreetly but also of ensuring the security and privacy of the digital images serving as the transmission medium.

Reversible data hiding (RDH) is a branch of data hiding, which emphasizes embedding secret data in a reversible way and allows the receiver to recover the original image. Lo and Hu(2014)^4^ proposed a digital image reversible authentication scheme that embeds the image’s block authentication code directly into the corresponding image block. The scheme(2016)^5^ advances upon this concept by amalgamating reversible data hiding with authentication technology, thereby safeguarding the integrity of images with specific applications such as in medical and military contexts. Additionally, RDH based on deep learning^6–8^ has been extensively researched, and significant improvements have been made in terms of embedding capacity. However, it is still impossible for most deep learning-based steganography techniques to achieve full reversibility of carrier images.

In recent years, in order to protect the contents of the original image, researchers have proposed a large number of reversible data hiding algorithms using encrypted images as carriers. Reversible Data Hiding in Encrypted Images (RDHEI) algorithms are classified based on various emphasized aspects. The two main categories of these algorithms, based on the creation of embedding space, can be bifurcated into Vacating Room After Encryption (VRAE), which encrypts the original image before embedding, and Reserving Room Before Encryption (RRBE), which preprocesses the image to leave space before encryption and then encrypts the preprocessed image and perform embedding operation.

However, most of the existing RDHEI methods do not protect the integrity of the carrier image. In this paper, an improved prediction algorithm ISGAP is designed, which can get a result that is closer to the original value. Then a reversible data hiding and authentication scheme for encrypted image is proposed. Under the premise of ensuring large-capacity data secret transmission and reversible recovery of the original image, the authentication of plaintext image and ciphertext image can be realized. The integrity of the secret carrier, secret data, and plaintext carrier is protected.

The rest of this paper is organized as follows. In the second section, we review the related work. In the third section, we introduce the overall framework of the scheme and describe its details. In Sect. 4, we discuss the experimental results and the performance of the scheme. In Sect. 5, we summarize our scheme.

## Related work

In this section, we introduce the existing VRAE methods and RRBE methods.

### VRAE

In the VRAE scheme, the image needs to be encrypted first and then the data is embedded. Puech et al. (2008)^9^ proposed one of the initial VRAE schemes, their method first performs block encryption on the image and selects the embedded bit according to the data embedding key to embed the information. Wu and Sun (2014)^10^ designed an RDHEI method where the original image is first encrypted using a stream encryption algorithm, followed by data hiding through the selection of a pixel subset based on a data hiding key. Dragoi et al. (2017)^11^, Dragoi and Coltuc (2018)^12^ then enhanced the method in different ways. These methods do not achieve lossless recovery of image; hence they are not completely reversible. Puteaux and Puech (2018)^13^ encrypted the original image by replacing the most significant bit (MSB) of each available pixel in the encrypted image with a small amount of secret data.

All aforementioned methods rely on pixel correlation to create space, while several methods also proposed based on the correlation between pixel blocks to achieve space carving. Chen KM (2020)^14^ divided the encrypted image into non-overlapping blocks, calculating the differences between the reference pixels within blocks and other pixels. They then compressed these pixel differences to make room for hiding secret data. Liu and Pun (2020)^15^ utilized traditional XOR methods for block encryption, preserving redundancy in encrypted blocks. The redundancy inherent in some encryption blocks created space for secret data, with the direct decryption images of the scheme maintaining high quality. Wang and He (2021)^16^ successfully compressed blocks using the same MSB of the pixels within the block, though this approach proved space-consuming in complex images. Gao et al. (2022)^17^ segmented the image into blocks and encrypted it using block replacement and block-level stream cipher. The data hider then analyzes the blocks and adaptively determines the best block type label for encoding, compressing the image to obtain the space needed to embed the encrypted secret data.

### RRBE

In the RRBE approach, the content owner makes space in the plaintext image by preprocessing it before the image is encrypted. Ma et al. (2013)^18^ proposed the first RRBE method, which embeds the least significant bit (LSB) of some pixels in the complex region into the smooth region, thereby reserving the LSB space of some pixels for embedding data. Zhang et al. (2014)^19^ improved the scheme^18^ by moving the histogram of some prediction error pixels before encrypting the image to release space for data embedding. The embedding rate and peak signal-to-noise ratio (PSNR) of the decrypted image are improved. Chen et al. (2014)^20^ introduced RDHEI method using the Paillier cryptosystem, segmenting each pixel of the original image into two parts: the 7 MSB and the 1 LSB, allowing separate encryption and one bit of the message is embedded into each pair of adjacent pixels. Shiu et al. (2015)^21^ implemented differential expansion within homomorphic encryption domains, effectively resolving the insufficient/overflow challenges. Another method^22^ used a small number of pixels as reference values to calculate the prediction errors of most pixels and uses a parametric binary tree labeling method to distinguish prediction errors.

Several high capacity reversible data hiding algorithms are proposed; Chen and Chang (2019)^23^ proposed a bit-plane compression algorithm. They compress the MSB plane of the image to generate space for high-capacity embedding. Yin et al. (2019)^24^ adaptively predicted multiple MSB for each pixel, and marked the original image with Huffman coding, and encrypted the image with stream cipher. Through multiple MSB replacements, the freed space can be used to embed additional data. This method achieves high embedding capacity. Rai et al. (2023)^25^ first calculates the prediction errors using GED predictor and then creates a room inside the original image by encoding them using Huffman coding. Finally, the image is encrypted using stream-cipher and the reserved room is used to embed the secret payload. In (2023)^26^, the prediction errors first partition and then compressed by using Huffman coding, which frees up a considerable amount of space. Additionally, adopt a multi-level embedding principle, resulting in a significant increase in capacity. Yao et al. (2023)^27^ compresses global zero-valued high bit-planes block-wise and adaptively allocates Huffman indicators based on occurrence frequency, significantly increasing the embedded payload. Zhang et al. (2024)^28^ classifies pixel blocks by counting consecutive consistent value bit-planes from top to bottom, allowing each pixel block to embed data and exploring a larger data embedding space.

The VRAE and RRBE methods previously mentioned fail to maintain the integrity of the image, which leaves the receiver uncertain about the authenticity of the secret image, whether it has been tampered with or not. This paper proposes a reversible data hiding and authentication scheme for encrypted images to implement reversible recovery and content authentication of cover images and secret data.

### Proposed scheme

The proposed scheme involves three parties: the content owner, the data hider, and the receiver. The content owner employs an enhanced predictor ISGAP to estimate the pixels of the carrier image, generating prediction errors which are then compressed to create a substantial amount of space for data embedding. Utilizing hash algorithms and digital signature algorithm in tandem, the content owner generates authentication data for the original image—composed of a hash value, *hash*1, and a signature, *SIG*1—which is then embedded in the reserved space, yielding a preprocessed image that is subsequently encrypted with stream cipher to create an encrypted image, ultimately transferred to the data hider.

The data hider encrypts the secret data intended for embedding using a stream cipher, then finds the reserved space’s location information from the encrypted image, embeds the encrypted secret data into these reserved space, thereby obtaining a semi-marked encrypted image, which is then subjected to a hash algorithm and digital signature algorithm to generate the authentication information—*hash*2 combined with *SIG*2—of the semi-marked encrypted image, conclusively embedding this ciphertext authentication information to produce the marked encrypted image.

**Fig. 1:**
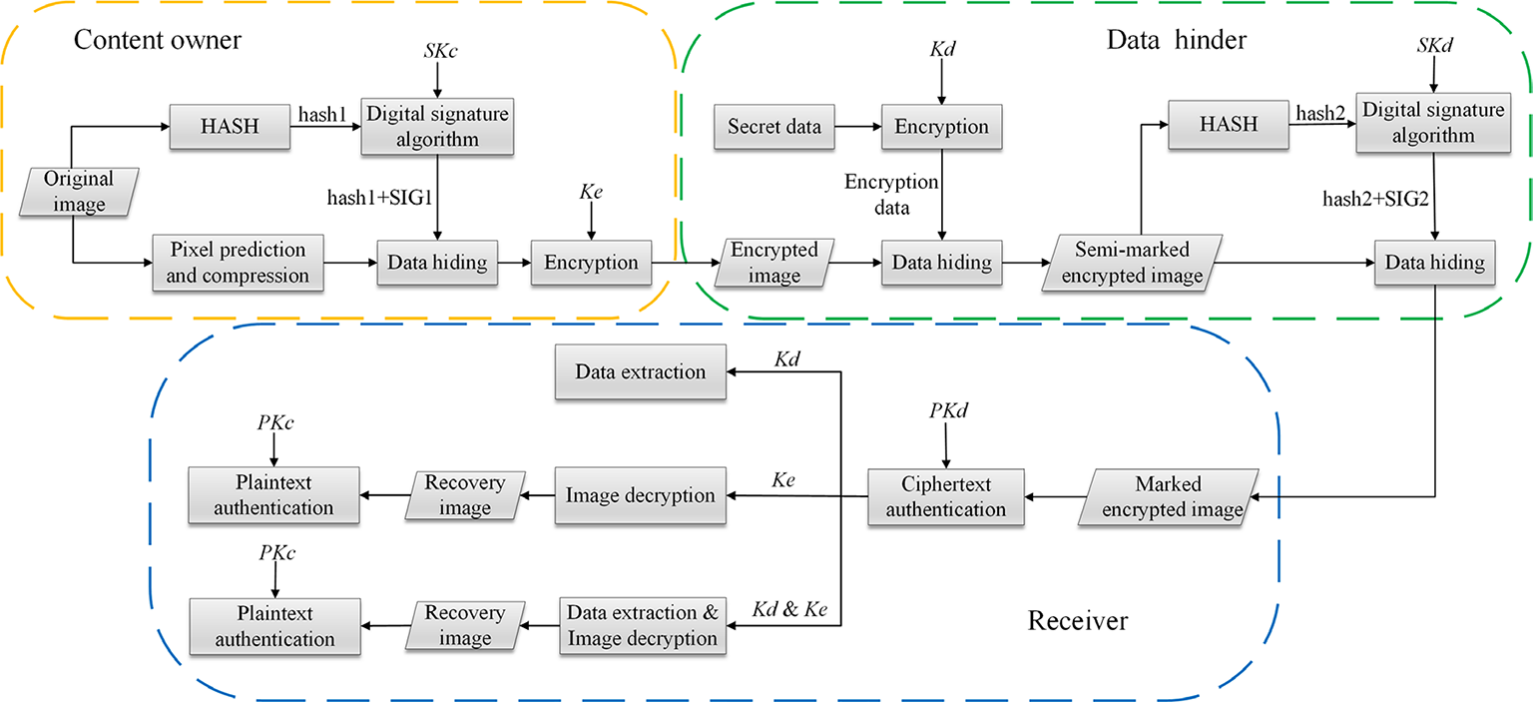
Framework of the proposed scheme.

After receiving the marked encrypted image, the receiver performs ciphertext authentication firstly. After the authentication is successful, the receiver can extract data or recover the original image according to different keys and perform plaintext authentication. The framework of this scheme is shown in Fig. 1 and (*SKc*、*PKc*) represents a pair of public and private keys of the content owner, while (*SKd*、*PKd*) is a pair of public and private keys of the data hider. *Ke* is the image encryption key, and *Kd* is the data hiding key.

### The improvement of prediction

This section presents an improved prediction method which can predict the pixel value more accurately, so that the prediction error is concentrated in a small range, providing higher embedding capacity.

#### SGAP

**Fig. 2:**
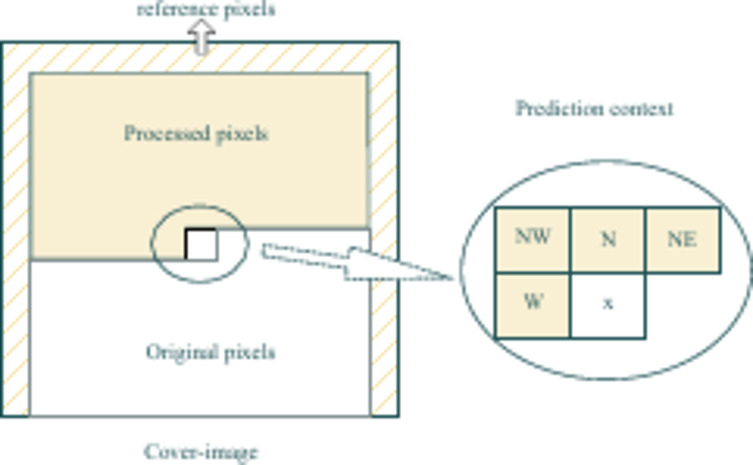
SGAP.

The gradient adjusted predictor (GAP) is an adaptive predictor that adjusts based on seven different adjacent pixels around the predicted pixel. Its simplified version, known as SGAP, provides almost similar results to GAP, but with lower computational costs. It uses four adjacent pixels of the target pixel *x*: the left pixel (*x*_*W*_), the upper left pixel (*x*_*NW*_), the upper pixel (*x*_*N*_), and the upper right pixel (*x*_*NE*_) to predict its pixel value, as shown in Fig. 2. The pixels in the first row, first column, and last row are used as reference pixels and remain unchanged during the prediction process.

Equation (1) can be used to calculate the predicted value *px* for pixels in the cover image except the reference pixel.

1 $$px=\frac{{{x_N}+{x_W}}}{2}+\frac{{{x_{NE}} - {x_{NW}}}}{4}$$

#### ISGAP

**Fig. 3:**
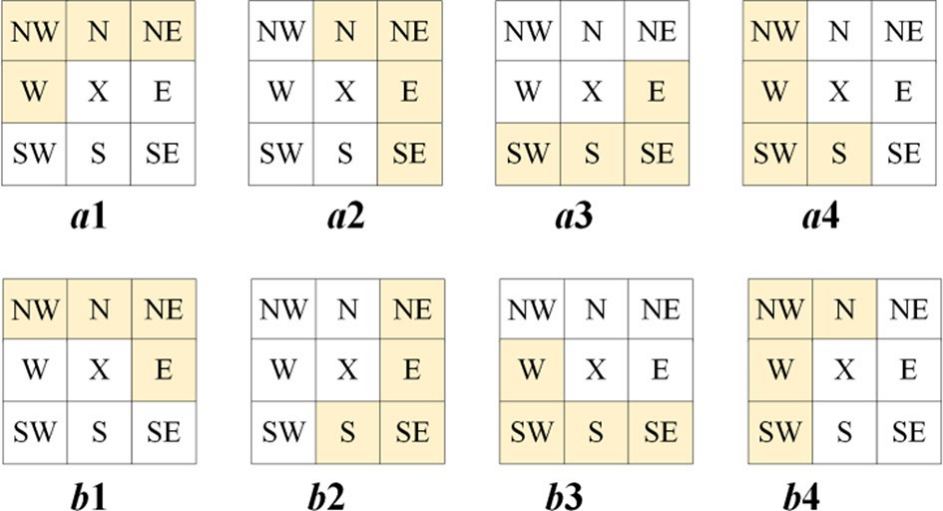
Eight models of ISGAP.

To enhance the accuracy of prediction, the improved SGAP, called ISGAP, supplements the SGAP with 7 new models, as shown in Fig. 3. ISGAP employs four edge pixels (the first and last row, first and last column) of original image as reference points. Users are able to select one out of eight models which yields highest accuracy for predicting images.

The equation for predicting images in eight models of ISGAP is different. Model *a*1 is the same as the original SGAP, and the calculation method is shown in Eq. (1). The prediction equation for model *b*1 is shown in Eq. (2).

2 $$px=\frac{{{x_N}+{x_E}}}{2}+\frac{{{x_{NW}} - {x_{NE}}}}{4}$$

Equation (3) shows the prediction equations of other models.

3 $$\begin{gathered} a2:px=\frac{{{x_N}+{x_E}}}{2}+\frac{{{x_{SE}} - {x_{NE}}}}{4}{\text{ }}b2:px=\frac{{{x_S}+{x_E}}}{2}+\frac{{{x_{NE}} - {x_{SE}}}}{4} \hfill \\ a3:px=\frac{{{x_S}+{x_E}}}{2}+\frac{{{x_{SW}} - {x_{SE}}}}{4}{\text{ }}b3:px=\frac{{{x_S}+{x_W}}}{2}+\frac{{{x_{SE}} - {x_{SW}}}}{4} \hfill \\ a4:px=\frac{{{x_S}+{x_W}}}{2}+\frac{{{x_{NW}} - {x_{SW}}}}{4}{\text{ }}b4:px=\frac{{{x_N}+{x_W}}}{2}+\frac{{{x_{SW}} - {x_{NW}}}}{4} \hfill \\ \end{gathered}$$

### Content owners

The content owner performs the following operations in turn: (1) Using ISGAP to predict the pixel value, generating a prediction error matrix that is then partitioned into blocks. (2) Merging and encoding block types. (3) Compressing the error block and embed plaintext authentication information to create a preprocessed image. (4) Encrypting the preprocessed image.

#### Pixel prediction

When using ISGAP to predict original image *I*_*O*_, the four edge pixels that serve as reference pixels are denoted as *Pr.* For pixels *x*_*i, j*_, assuming that the predicted value is represented as *px*_*i, j*_. All *px*_*i, j*_, and reference pixels *Pr* form the predicted image *I*_*p*_. Equation (4) is used to calculate the prediction error *e*_*i, j*_ for each pixel except *Pr*, where (2 ≤ *i* ≤ *M-*1,2 ≤ *j* ≤ *N*−1).

4 $${e_{ij}}={x_{ij}} - p{x_{ij}}$$

After removing reference pixels, all prediction errors form an error matrix of size (*M*−2)×(*N*−2). For original image *I*_*O*_, eight different prediction error matrices can be formed using ISGAP. Calculate the sum of absolute values of all elements in each error matrix according to Eq. (5), where *k*∈{*a*1, *a*2, *a*3, *a*4, *b*1, *b*2, *b*3, *b*4}. The prediction model with the smallest value is selected as the final prediction result of ISGAP predictor.

5 $$Absumy(k)=\sum\limits_{{i=2}}^{{M - 2}} {\sum\limits_{{j=2}}^{{N - 2}} {{e_{ij}}} }$$

**Fig. 4:**
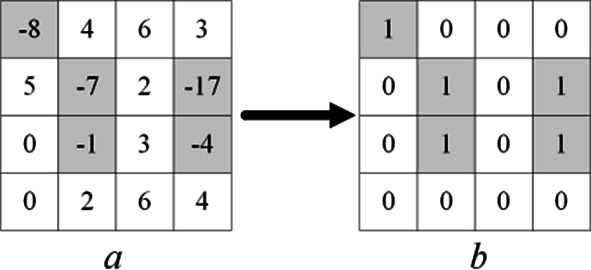
The example of generating a location map.

The prediction error matrix generated by ISGAP predictor is denoted as *P*, and a location map with the same size is generated to record positive and negative prediction errors, denoted as *P*-map. Figure 4 illustrates an example, *a* is the prediction error matrix and *b* is the *P*-map.

The absolute values of the elements in error matrix *P* are computed to obtain matrix *P*1. The prediction error image, composed of edge pixels *Pr* and matrix *P*1, is denoted as *I*_*OP*1_. The content owner partitions the error matrix *P*1 into *n* non-overlapping blocks *B*_*i*_ (*i* = 1, 2., *n*) of size *s×s*, where *n=*⌊(*M*−1)×(*N*−2)/(*s*×*s*)⌋. Each block contains *s*^2^ prediction error values represented by *e*_*t*_, where *t =* 1,2. *s*^2^. The first error pixel *e*_1_ in each block serves as the reference pixel.

#### Block type merging and encoding

For each matrix block *B*_*i*_, the most significant bit (MSB) to the least significant bit (LSB) of the reference error value *e*_1_ and the remaining *s*^2^*-*1 prediction error *e*_*t*_ in the block are compared successively until a certain bit is different. Let *α* represent the length of the same sequence of bits in all error values in the block, called the flag bit. After that the content owner can determine the block type corresponding to each matrix block, where *α*∈{0,1,2,., 8}.

**Fig. 5:**
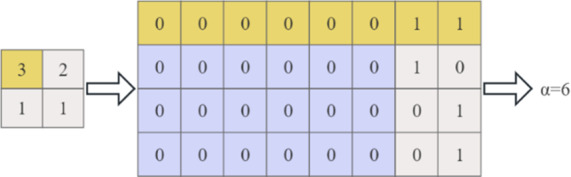
The example of 2 × 2 error matrix block.

As shown in Fig. 5, for a matrix block of size 2 × 2, the reference error pixel is 3, After converting all the remaining error pixels into 8-bit binary sequences, and comparing their MSB to LSB in turn, only the two least significant bits differ. Thus, this matrix block type value *α* = 6.

To recover the original image, it is imperative to record each error block’s complete information, which entails documenting the type value *α*, the reference error pixel value *e*_1_, and the (8 − *α*) distinct least significant bits (LSB) of the remaining *s*^2^ − 1 prediction errors for full error block reconstruction. We use block type merging and Huffman coding to adjust the amount of data used to record a label value. Assuming that the amount of data required to record a type value is consistent, the smaller *α* is, the less space is left. For enhanced embedding capacity, an ideal distribution would have a reduced count of blocks with smaller *α* and an increased count with larger *α* values.

**Fig. 6:**
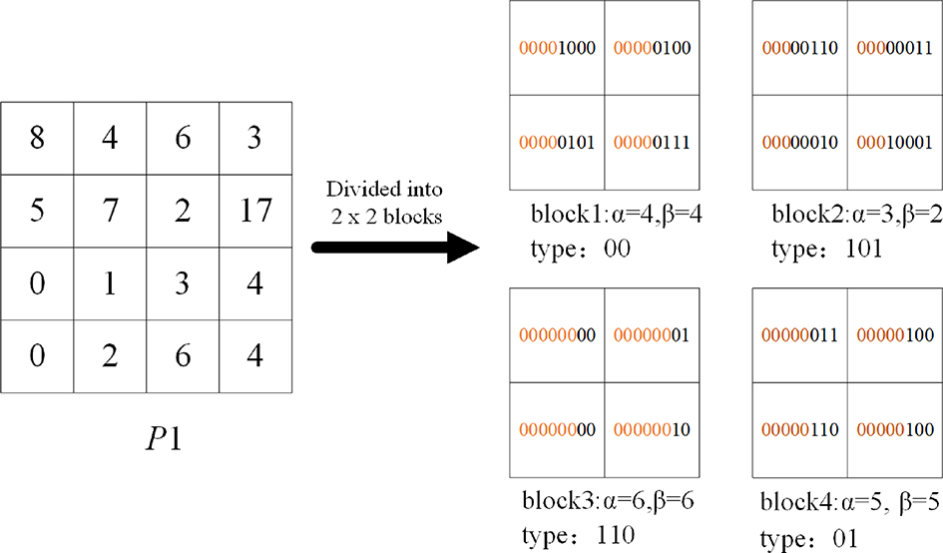
The example of type encoding.

Block types are merged into six types to reduce the storage required to embed type values. Use *β* to denote the value of the merged block type, *β*∈{*β*_*s*_, *β*_*c*_}. Let *β*_*s*_ = 0,2,4,5,6,7 for the smooth image, and the types 0,1 are merged into 0; the types 2,3 are merged into 2; the types 7,8 are merged into 7; other types do not change. For complex images, the combined block type values *β*_*c*_ = 0,2,3,4,5,7. Six fixed-length Huffman codes {00,01,100,101,110,111} are used to adaptively encode the six merged block types, where the shorter code marks the type value with the larger number of blocks and the longer code marks the type value with the fewer number of blocks. Figure 6 shows an example of type encoding for a smooth image.

#### Preprocessed image generation

To generate the preprocessed image, the content owner first generates the plaintext authentication information, and then compresses the error matrix *P*1 to reserve space. The center matrix *P*2 of the preprocessed image is constructed by using the plaintext authentication information, the compressed bit-streams of *P*1 and the auxiliary information.

Step 1: The content owner generates the hash value *hash*1 of original image by using the hash algorithm and signs *hash*1 to obtain *SIG*1 using the private key *SK*_*c*_.

Step 2: Compress the prediction error matrix *P*1. For each block in *P*1, content owner records the type of the block, the value of the prediction error at the first position, and the (8 − *β*) least significant bit of the remaining prediction error within the block. After all prediction error blocks are processed, the compressed bit-streams can be obtained.

**Fig. 7:**
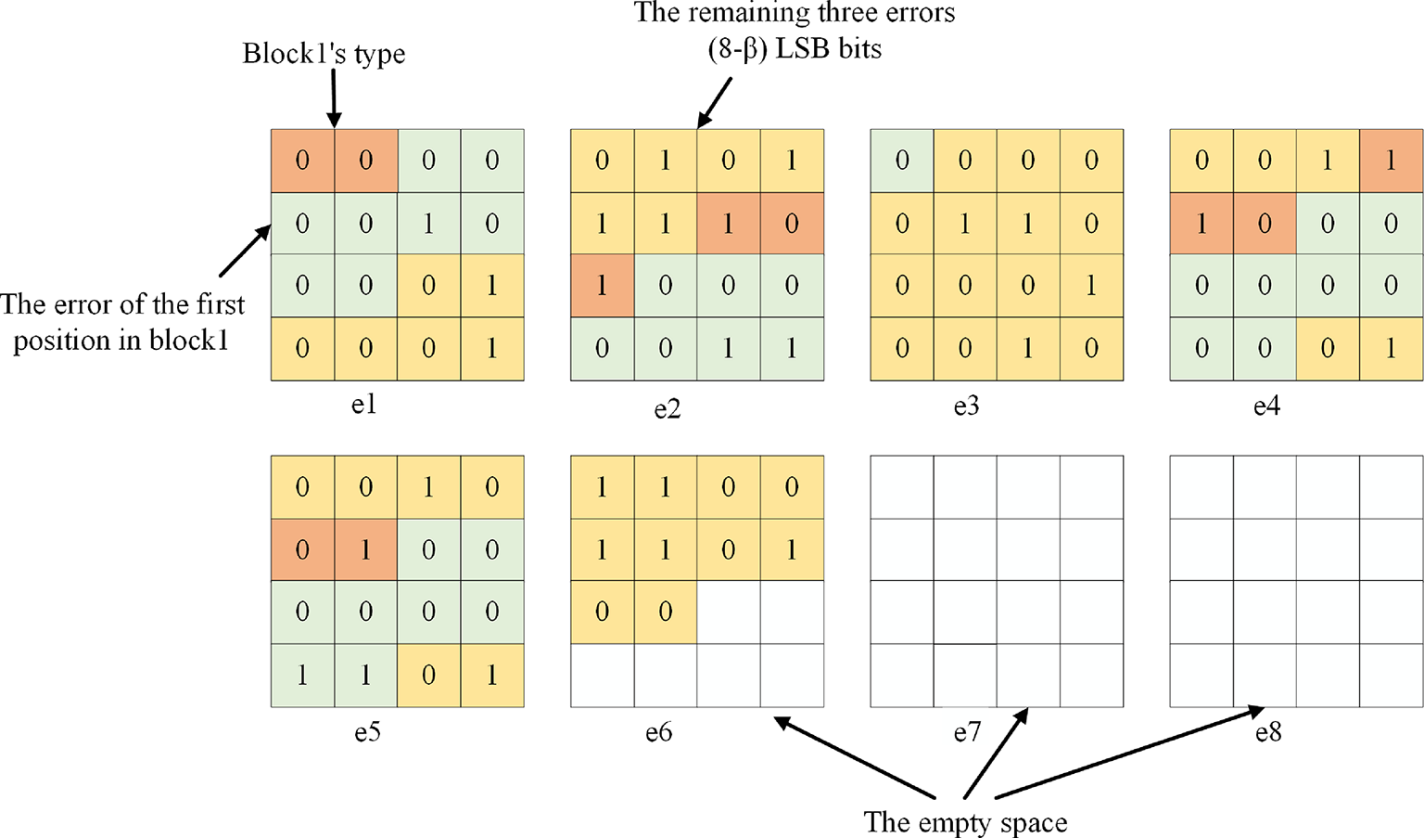
The bit-streams of *P*1 after compression.

In Fig. 7, the compressed bit-streams of *P*1 are placed from the highest bit plane to the lowest, and the blank part is the free space, where e1 ~ e8 represents 8-bit planes.

Step 3: Construct the preprocessed image.

Placing the following information at the multi-MSB planes of *P*1:

(1) The prediction model and Huffman coding dictionary.

(2) The compressed bit-streams of *P*1 and auxiliary information (includes *P*-map and overflow pixels and their location).

(3) The plaintext authentication information(*hash*1 + *SIG*1).

**Fig. 8:**
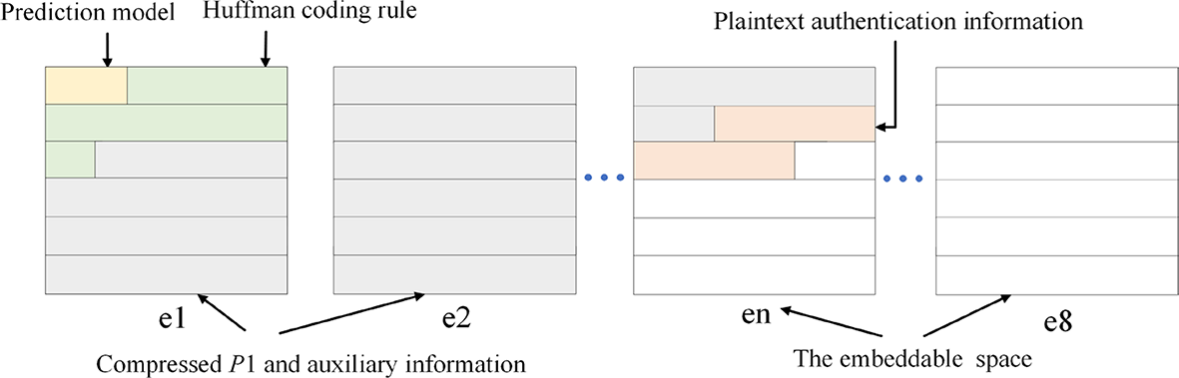
The bit planes distribution of *P*2.

After placing this information, the vacated multi-LSB planes are complemented by 0 to obtain the center matrix *P*2. The preprocessed image, composed of edge pixels *Pr* and matrix *P*2, is denoted as *I*_*OP*2_. Figure 8 illustrates the bit plane information of the central matrix *P*2.

#### Image encryption

The content owner generates an encrypted image *I*_*e*_ by following steps:

Step1: A pseudo-random bit sequence *Ke* of the same size as the image is generated.

Step2: Using *Ke* encrypt the preprocessed image *I*_*OP*2_ bit by bit to generate the encrypted image *I*_*e*_.

After encryption, the content owner places the starting position information of the embeddable space in the LSB plane of *I*_*e*_ and sends it to the data hider.

### Data hider

After receiving the encrypted image, the data hider first obtains the starting position, and then encrypts the secret data. The encrypted secret data is then embedded into the reserved space according to the starting position to generate the semi-marked encrypted image, which is processed to generate ciphertext authentication information. The ciphertext authentication information is then embedded in the semi-marked encrypted image to generate marked encrypted image.

#### Secret data embedding

Step 1: Extracting the start position information of the embeddable space from *I*_*e*_.

Step 2: Using the data hiding key *K*_*d*_ encrypts the secret data to obtain the encrypted secret data.

Step 3: Embedding the encrypted secret data to the reserve space of *I*_*e*_. The remaining space is denoted as *L* and its bits are set to 0 for embedding ciphertext authentication information, so we obtain the half-marked encrypted image *I*_*eh*_.

**Fig. 9:**
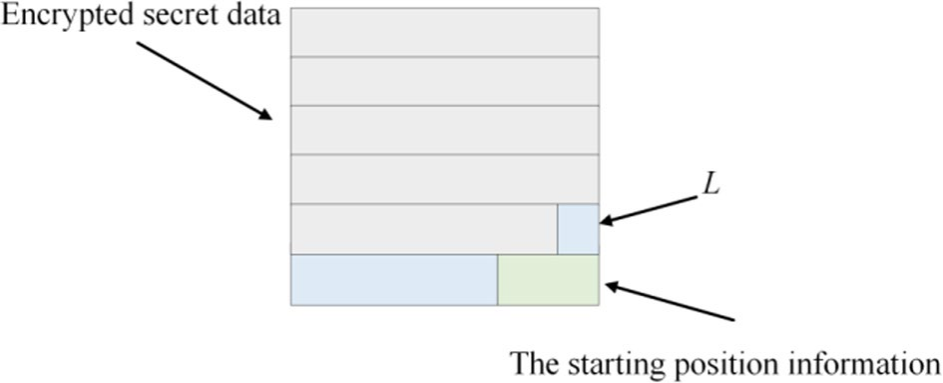
illustrates the LSB plane distribution of *I*_*eh*_.

Figure 9 The LSB plane distribution of *I*_*eh*_ (except the edge pixels).

#### Ciphertext authentication information embedding

The data hider uses the hash algorithm to process *I*_*eh*_ and generate the hash value *hash*2. The *SIG*2 is obtained by signing *hash*2 with the private key *SK*_*d*_. Subsequently, the data hider embeds the ciphertext authentication information (*hash*2 + *SIG*2) into *L* to obtain the marked encrypted image *I*_*ec*_.

### Receiver

After receiving the marked encrypted image *I*_*ec*_, the receiver can first extract the ciphertext authentication information in the space *L* and then use *PK*_*d*_ to execute ciphertext authentication:

Step 1: Verify whether the ciphertext authentication information is legitimate using the public key *PK*_*d*_.

Step 2: Set the space *L* of *I*_*ec*_ to 0, and generate the hash value, which is compared the hash value extracted from *L* to realize ciphertext authentication.

When the ciphertext authentication is passed, different functions can be realized according to different keys.

(1) The receiver only has *K*_*e*_.

The original image can be recovered, and plaintext authentication can be performed. Firstly, the marked encrypted image *I*_*ec*_ can be decrypted by *K*_*e*_. According to the starting position information, receiver can obtain *P*2, which includes the prediction model, Huffman coding rule, compressed *P*1 and auxiliary information, plaintext authentication information, combined with the reference pixels the original image *I*_*O*_ is gradually recovered.

After obtaining the recovered image, it can be authenticated:

Step 1: Verifying the plaintext authentication information using the public key *PK*_*c*_.

Step 2: Realizing the plaintext authentication by comparing the hash value in the plaintext authentication information with the hash value generated by *I*_*O*_.

(2) The receiver only has *K*_*d*_.

According to the starting position information, the receiver extracts the encrypted data, and then uses *K*_*d*_ to decrypt the encrypted data to obtain the secret data.

(3) The receiver has both *K*_*e*_ and *K*_*d*_.

The receiver can extract the data and recover the original image lossless and realize plaintext authentication.

## Experimental results and discussion

**Fig. 10:**
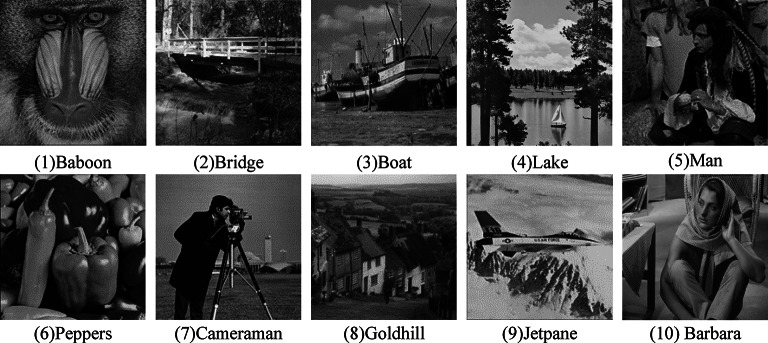
Test images.

o validate the security and efficiency of the proposed scheme, performance tests were conducted using a set of ten 512 × 512 grayscale images, including Baboon, Bridge, Boat, Lake, Man, Peppers, Cameraman, Goldhill, Jetpane, and Barbara, as depicted in Fig. 10. Furthermore, the comparative performance analysis of various methodologies was executed on two extensive image databases, namely BossBase^29^ and BOWS-2^30^.

The embedding rate (ER) is measured in *bpp* and is calculated as follows, where EC is embedding capacity (in *bits*).

6 $$ER=\frac{{EC}}{{M \times N}}$$

In addition, the Peak Signal-to-Noise Ratio (PSNR) defined by Eq. (7) and Structural Similarity (SSIM) defined by Eq. (9) are used to estimate the visual quality of the recoverd image and the original image, where *µ* is the mean, *σ* is the variance, and *c*1, *c*2 are constants.

7 $$PSNR=10{\log _{10}}\frac{{{{(255)}^2}}}{{MSE}}dB$$

8 $$MSE=\frac{1}{{M \times N}}\sum\limits_{{i=1}}^{M} {\sum\limits_{{j=1}}^{N} {{{({x_{i,j}} - {y_{i,j}})}^{^{2}}}} }$$

9 $$SSIM((x,y)=\frac{{(2{\mu _x}{\mu _y}+{c_1})(2{\sigma _{xy}}+{c_2})}}{{(\mu _{x}^{2}+\mu _{y}^{2}+{c_1})(\sigma _{x}^{2}+\sigma _{y}^{2}+{c_2})}}$$

### Security analysis

#### Shannon information entropy

Shannon information entropy can be used to measure the randomness of the information source. The maximum Shannon information entropy of gray image is 8, and the equation is as follow, where *P*_*i*_ is the probability that a pixel gray value occurs in that image.

10 $$H(E)= - \mathop \sum \limits_{{i=0}}^{{255}} {P_i}\log {P_i}{\text{ }}$$

Table 1 is obtained by experiments on several standard images in Fig. 10. The information entropy of the preprocessed image is higher than that of the original image, which is because the pixel information of the image is rearranged and scrambled for the compression process. The entropy of the encrypted image tends to the maximum, which indicates that the security of the algorithm can be guaranteed.

**Table 1:** Comparison of information entropy.

Test image	Original image	Preprocessed image	Encrypted image
Barbara	7.4664	7.8006	7.9993
Baboon	7.3579	7.7853	7.9993
Cameraman	7.0478	7.6508	7.9993
Goldhill	7.4778	7.7669	7.9993
Man	7.3574	7.7352	7.9994

#### Key-space

By analyzing the key space, we can get the probability of obtaining the original image without the image encryption key *K*_*e*_. For an *M* × *N* preprocessed image, we encrypt it with a pseudo-random binary sequence of length *M* × *N* × 8, and each bit in the sequence is likely to be 0 or 1. In other words, this pseudo-random sequence has a total of 2^*M*×*N*×8^ possibilities, which is a large number. So in the absence of the *K*_*e*_, it is almost impossible to obtain a completely correct encryption sequence from so many possibilities. This proves that our method is highly secure, which protects the privacy of the content owner.

#### Single image test

**Fig. 11:**
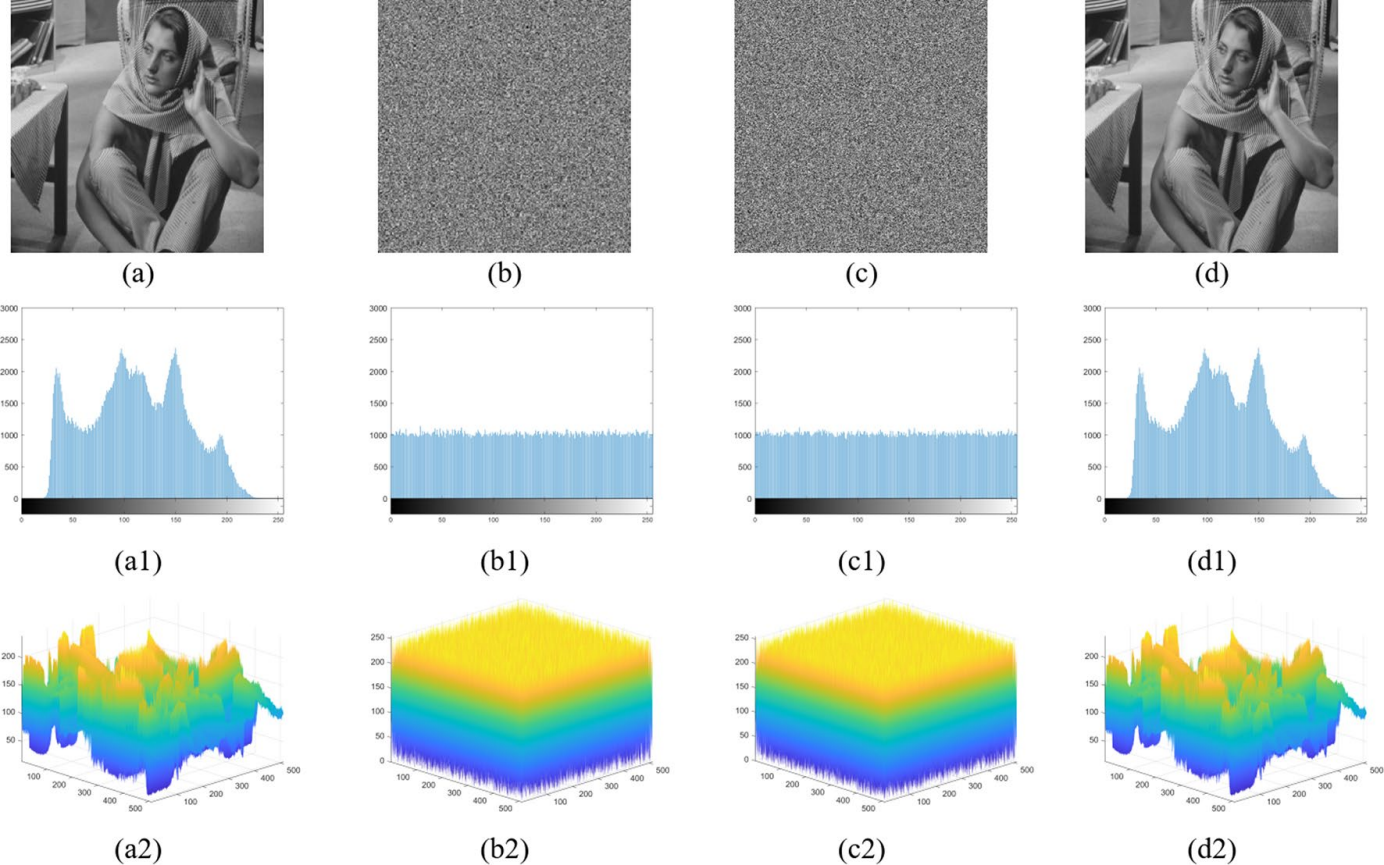
(**a**) Original image; (**b**) Encrypted image;(**c**) Marked encrypted image with the ER = 2.354bpp; (**d**) Recovered image. (a1-d1) Histogram distribution of the images a-d. (a2-d2) Pixel value distributions of the images a-d.

Fig. 11 takes the grayscale image Barbara of size 512 × 512 and a block size of 4 × 4 as an example. (a) shows the original image, (b) the encrypted image, (c) the marked encrypted image with the ER = 2.74 bpp, and (d) the recovered image. (a1) to (d1) show the corresponding histograms, and (a2) to (d2) show the corresponding pixel value distributions. From (a1) to (c1), both the encrypted image and the marked encrypted image have a completely different histogram distribution compared to the original image. From (a2) to (c2), it is difficult to identify any content of the original image from the encrypted or marked encrypted image using statistical analysis. By comparing the histograms and pixel value distributions of the original image (a) and the recovered image (d), it is found that PSNR = ∞ and SSIM = 1, indicating that the scheme is reversible.

### Performance analysis

In this section, we analyze the performance of the ISGAP and the embedded performance under different parameters. We compare the performance of SGAP and ISGAP in 6 complex images and 4 smooth images, respectively.

#### Predictor performance analysis

The Mean Absolute Error (MAE) is a commonly used measure of the difference between the predicted value and actual value, and the equation is as follow, where *n* is the number of samples, *y*_*true* is actual value, and *y*_*pred* is the predicted value.

11 $$MAE=\frac{1}{n}\sum\limits_{{y=1}}^{n} {|y\_true{\text{ }} - {\text{ }}y\_pred|}$$

**Table 2:** Comparison of mean absolute error values (MAE).

Test image(512 × 512)	SGAP	ISGAP	Percentage reduction
Baboon	14.344	13.687	4.58%
Bridge	10.352	10.007	3.33%
Boat	6.404	6.356	0.75%
Cameraman	2.739	2.739	0
Barbara	8.300	8.288	0.14%
Jetplane	3.858	3.775	2.15%
Man	6.972	6.880	1.32%
Peppers	4.893	4.834	1.21%

A lower MAE value indicates better predictive performance, Table 2 displays the MAE of prediction errors after employing SGAP and ISGAP for predicting eight images, In seven out of eight images, ISGAP demonstrates superior prediction effectiveness compared to SGAP. We also conducted tests on two datasets, where the average reduction in MAE on the BossBase dataset was 5.97%, with a maximum reduction of 35.9%. On the BOWS-2 dataset, the average reduction was 7.36%, with a maximum reduction of 30.4%. The decrease in MAE can be attributed to the ISGAP model’s ability to predict from multiple directions, which is more accurate than single-direction prediction. This highlights ISGAP’s advantage in minimizing prediction errors. This significant improvement demonstrates that ISGAP provides a more accurate model for further large-capacity data hiding.

**Fig. 12:**
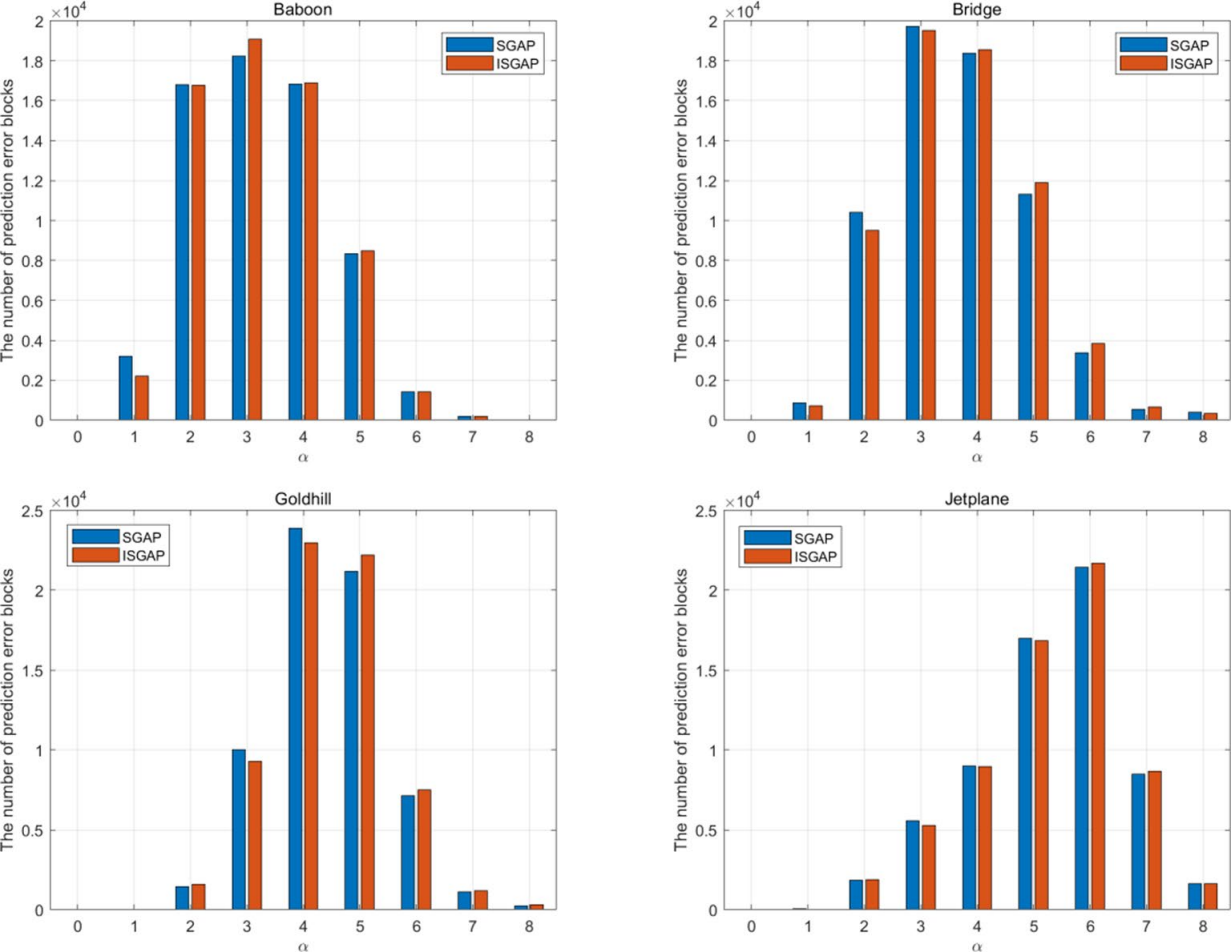
Block distribution of prediction errors generated by SGAP and ISGAP in nine different block types (α)

Table 3 shows the optimal prediction mode of all the images in Fig. 10 when ISGAP is used for prediction. It can be seen that only one image has the optimal prediction result using *a*1 mode. Therefore, the performance of SGAP is generally inferior to ISGAP. Figure 12 shows the block distribution (*α*) of the error blocks generated by SGAP and ISGAP prediction in nine different block types for four images. It can be seen that the number of prediction error blocks generated by ISGAP is more than that generated by SGAP when *α* is big. So, ISGAP predictor can save more space. In addition, we performed experiments on more images and obtained similar results.

**Table 3:** Optimal modes of ISGAP prediction for different images.

Image	Baboon	Bridge	Boat	Lake	Man	Peppers	Cameraman	Goldhill	Jetplane	Barbara
Mode	*a*4	*b*1	*b*4	*a*2	*b*2	*a*3	*a*1	*b*3	*a*2	*a*3

#### Embedding rate under different parameters

Table 4 shows the ER between the proposed scheme and the scheme proposed by Ren et al. (2023) in 10 images with different block sizes. As can be seen from Table 4, the ER of the proposed method is generally higher than that of Ren et al. (2023), mainly because ISGAP’s prediction results are better than SGAP’s. In addition, the scheme adopts Huffman encoding for adaptive label values, which reduces the space required to record label values, this further enhances the embedding capability.

**Fig. 13:**
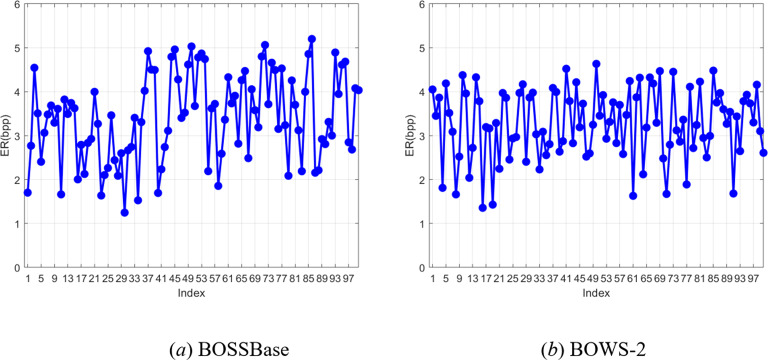
ER for 100 randomly selected images from BOSSBase and BOWS-2.

We also tested the embedding rate of the proposed scheme by selecting 100 images from two image datasets, BOSSBase and BOWS-2, respectively, as shown in Fig. 13. For most of the test images, the proposed method achieves the highest ER when the block size is *s* = 4 or =5. As shown in Table 5, the highest embedding rates of the proposed method on the BOSSbase and BOWS-2 can reach 5.20bpp and 4.63bpp, respectively. The average embedding rates on the two datasets are more than 3bpp, and the PSNR of all the recovered images is + ∞ and SSIM is 1.

**Table 4:** The ER of the proposed scheme and Ren et al. (2023) of different images under different block sizes.

Image	s = 2		s = 3		s = 4		s = 5		s = 8	
	[Ren]	Proposed	[Ren]	Proposed	[Ren]	Proposed	[Ren]	Proposed	[Ren]	Proposed
Baboon	0.751	0.793	1.143	1.218	1.134	1.238	1.076	1.177	0.767	0.886
Bridge	1.081	1.125	1.530	1.604	1.528	1.616	1.485	1.579	1.137	1.242
Boat	1.526	1.589	2.150	2.155	2.226	2.229	2.228	2.236	1.968	1.980
Lake	1.500	1.554	2.106	2.107	2.177	2.186	2.159	2.191	1.957	1.957
Man	1.461	1.525	2.046	2.053	2.078	2.082	2.057	2.063	1.719	1.739
Peppers	1.825	1.828	2.352	2.452	2.366	2.527	2.316	2.539	1.941	2.259
Cameraman	2.635	2.635	3.538	3.577	3.666	3.686	3.717	3.732	3.469	3.477
Goldhill	1.599	1.639	2.073	2.130	2.064	2.137	2.023	2.098	1.688	1.768
Jetpane	2.199	2.265	2.895	2.938	2.964	3.036	2.977	3.024	2.647	2.740
Barbara	1.522	1.537	2.134	2.208	2.218	2.354	2.250	2.421	1.996	2.214
**average**	**1.610**	**1.649**	**2.197**	**2.244**	**2.242**	**2.310**	**2.229**	**2.306**	**1.929**	**2.026**

**Table 5:** Experimental results of the bossbase and BOWS-2.

Datasets	ER			PSNR	SSIM
	Best case	Worst case	Average		
BOSSbase	5.20	1.248	3.39	+∞	1
BOWS-2	4.63	1.357	3.27	+∞	1

### Comparison with existing methods

**Fig. 14:**
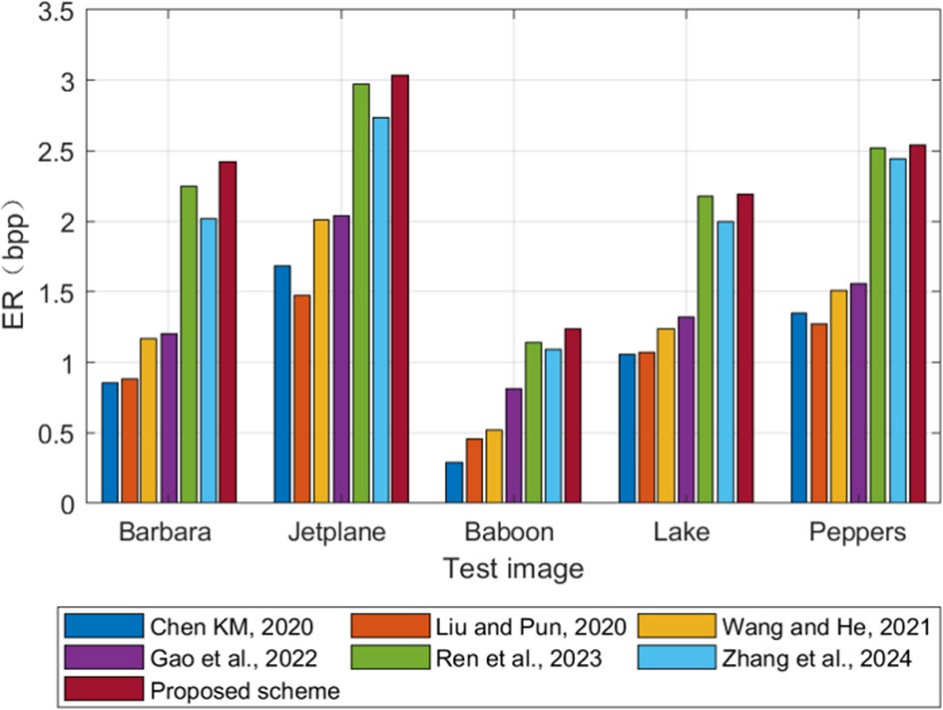
ER comparison with other five schemes.

The proposed scheme is compared with other six schemes^14–17,26,28^ in order to show the advantages of ER. As shown in Fig. 14, We selected five test images Barbara, Jetpane, Baboon, Lake and Peppers. For both Ren et al. (2023) and the proposed scheme, we adopt the block mode that maximizes the embedding capacity of each image. Compared to the VRAE methods^14–17^, the proposed scheme first performs image preprocessing based on the RRBE framework, which significantly compresses the image and utilizes the redundant space. Compared to the block-level processing RRBE methods^26,28^, it generates smaller prediction errors during the preprocessing stage using ISGAP. Furthermore, during the processing of error blocks, adaptive Huffman coding is employed, creating a larger embedding space.The results show that the ER of the proposed scheme is better than other schemes.

In order to test the ability of the adaptive Huffman coding for compression efficiency, We tested the 8 standard grayscale images in Fig. 10. Equation (12) calculates their compression rates (*C*_R_), where *C*_a_ and *C*_b_ represent the image’s size before and after compression, respectively. A larger *C*_R_ indicates more available space in the image for embedding secret information, leading to a higher embedding rate.

12 $${C_R}=\frac{{{C_{\text{a}}}}}{{{C_b}}}$$

The *C*_R_ obtained using the proposed scheme with optimal parameters are compared with those of existing lossless compression algorithms, including JPEG-LS, PNG, and Ren et al. (2023), as presented in Table 6. The data in the table shows that the proposed scheme achieves a higher compression ratio in most cases.

**Table 6:** Compression ratio comparison with other three schemes.

Test image	JPEG-LS	PNG	[Ren]	Proposed
Baboon	1.241	1.289	1.424	1.448
Bridge	1.394	1.624	1.547	1.569
Boat	1.411	1.516	1.783	1.785
Cameraman	1.879	2.276	2.740	2.766
Barbara	1.302	1.514	1.788	1.805
Jetplane	1.712	1.891	2.188	2.219
Goldhill	1.489	1.643	1.747	1.848
Peppers	1.556	1.657	1.891	1.929
Average	1.498	1.676	1.889	1.921

In addition, as show in Table 7, the proposed algorithm is compared with the existing schemes^3,4^, VRAE-based schemes^16,17^, and RRBE-based schemes^26–28^ in predictor and authentication.

Because of the use of ISGAP in proposed scheme, the embedding capacity is increased, and there is enough space for double authentication.

**Table 7:** Comparison with other seven schemes.

RDHEI Schemes	Predictors	Encoding	Encryption	Plaintext Authentication	Ciphertext Authentication
[Tew, 2016]	-	-	-	Yes	-
[Lo, 2014]	-	-	-	Yes	-
[Wang, 2021]	MED	Yes	Yes	-	-
[Gao, 2022]	MSB	Yes	Yes	-	-
[Ren, 2023]	SGAP	Yes	Yes	-	-
[Yao, 2023]	MED	Yes	Yes	-	-
[Zhang, 2024]	-	Yes	Yes	-	-
Proposed	ISGAP	Yes	Yes	Yes	Yes

### Analysis of experimental results

The proposed method ensures the integrity protection of both the cover image and ciphertext data while maintaining a relatively large embedding capacity. However, there are still some potential drawbacks and challenges with this approach.

During the experiments, for some images widely considered to be smooth, such as Jetplane, the embedding capacity obtained using smooth block-type merging was actually lower than that achieved with complex block-type merging. Therefore, we will continue to explore how to set parameters for appropriately selecting the block type based on the characteristics of each image, rather than simply categorizing them as complex or smooth. In terms of authentication, this paper implements the authentication of both the encrypted image and the embedded data as a whole. We aim to further explore the implementation of block-level authentication for the ciphertext image, which would enable tampering detection without decryption at the receiving end. However, handling the authentication information for each block without additional data transmission remains a challenge for us.

## Conclusion

In this paper, a reversible data hiding and authentication scheme for encrypted images based on prediction error compression is proposed. The improved ISGAP algorithm is used to generate smaller prediction errors, and the adaptive Huffman coding is used to compress error blocks to create more space. Combined with the bit plane rearrangement technology, the authentication information can be embedded into the ciphertext image, and the integrity authentication can be realized without additional transmission of authentication information. In testing on the BOSSbase and BOWS-2 datasets, the proposed ISGAP predictor achieves an average reduction in MAE of 5.97% and 7.36%, respectively, compared to the traditional SGAP predictor. The average embedding capacity reaches 3.39bpp and 3.27bpp, respectively, demonstrating that this approach offers an advantage in embedding capacity performance. Furthermore, the recovered images exhibit a PSNR = ∞ and SSIM = 1, ensuring perfect reversibility. The security and effectiveness of the scheme are validated through experimentation. In the future, we will further explore the characteristics of reversible data hiding for encrypted images to improve the algorithm’s performance and increase the functionality of the scheme.

## Data Availability

The datasets generated and analyzed during this study are available from the corresponding author on reasonable request.
